# Applications of Machine Learning Approaches in Emergency Medicine; a Review Article

**Published:** 2019-06-03

**Authors:** Negin Shafaf, Hamed Malek

**Affiliations:** 1 Faculty of Computer Science and Engineering, Shahid Beheshti University, Tehran, Iran.

**Keywords:** artificial intelligence, machine learning, emergency medicine, emergency service, hospital, triage

## Abstract

Using artificial intelligence and machine learning techniques in different medical fields, especially emergency medicine is rapidly growing. In this paper, studies conducted in the recent years on using artificial intelligence in emergency medicine have been collected and assessed. These studies belonged to three categories: prediction and detection of disease; prediction of need for admission, discharge and also mortality; and machine learning based triage systems. In each of these categories, the most important studies have been chosen and accuracy and results of the algorithms have been briefly evaluated by mentioning machine learning techniques and used datasets.

## Introduction

Artificial intelligence has been used in different health and medical fields. In particular, different artificial intelligence and machine learning techniques have attracted attention in the recent years. Emergency department (ED) and triage are among the most important parts of any hospital in which diagnostic and therapeutic interventions should be performed both rapidly and effectively. As the number of people referring to ED increases, common traditional techniques may not be sufficient. Thus, different methods of artificial intelligence such as natural language processing, data mining, clustering and classification algorithms should be used to significantly enhance the efficacy of hospital emergency system. Using artificial intelligence will bring some advantages; it will reduce human errors as well as time and expenses and improve the pace of providing services. Moreover, machine learning techniques usually have a comparable and even better accuracy compared with medical staff of the hospital.

Several studies have assessed application of artificial intelligence in ED triage from different points of view. Though, most of them can be divided into three categories, which will be further discussed in detail.

Machine learning systems have the potential of prediction and early detection of diseases in ED so that the disease can be treated more effectively and disease progression and occurrence of inappropriate complications can be prevented. This issue is discussed in detail in part 3.1 by reviewing the most important studies in this regard. Increase of the number of patients referring to ED will result in overcrowding, which decreases the efficiency of medical staff and increases the delay for patient visit. Consequently, artificial intelligence and machine learning techniques can be used in order to manage the ED, allocate money and equipment, and discharge patients more properly. A series of studies are provided in part 3.2 in this regard, in which admission of patients referring to ED, their discharge or mortality is predicated.

In part 3.3, studies on electronic triage methods based on machine learning techniques have been briefly reviewed. Studies presented in this part, prioritize patients based on different machine learning techniques, which is usually both faster and more accurate compared with traditional methods and emergency severity index (ESI) (1). Faster triage not only improves patient satisfaction, but also improves performance of ED and prevents its overcrowding.

**Machine learning techniques and its different types**

Machine learning is an important subset of artificial intelligence, which enables machines to learn and act on specific tasks. In fact, machine learning consists of a set of techniques and algorithms which can predict some future events or classify some data by learning the patterns in the existing data. Some of the most important algorithms in this field are logistic regression, support vector machines (SVM), Naive Bayes algorithm (2), decision trees, random forest, gradient boosting and deep learning. 

Logistic regression is a machine learning algorithm, which tries to find a linear model of the relations between variables by fitting a line on the curve of the given data (3). It can also be used for classification purposes.

One of the frequently used algorithms for data classification is SVM algorithm (4). It has been proven to find the best data classifier for the given data. SVM can achieve a very good generalization performance.

Decision tree is another tool for modeling data, which uses tree-like structures for classifying the decisions in order to output the class of the given data. When a number of decision trees are employed, an ensemble learning method called random forest is produced (5). 

Gradient boosting method is also widely used in some machine learning problems (6). It produces an ensemble model of the data by employing some weak models. One of the advantages of this method is its ability to reduce bias and variance in the model.

Deep learning is another algorithm, which has recently been widely recognized as a successful method in some complex machine learning tasks (7). Deep learning is a part of another class of machine learning methods named artificial neural network (ANN). In ANN algorithms, a network of cells is produced and the connections between the cells are adjusted in a way that the resulting network can learn the structure of the training data. In deep learning, the number of layers in the network is much higher than an ordinary ANN. This enables the algorithm to extract higher level features from the input data. 

**Review of studies**

In the following sections, the three most important issues in ED, including prediction and detection of disease; prediction of need to admission, discharge, and also mortality; and electronic triage will be discussed separately by reviewing the most important articles.

**Disease prediction and detection**

A lot of information about patient’s demographic characteristics, symptoms, and disease appearance is available in EDs through Electronic Health Record (EHR), nursing reports, laboratory test results and patient profiles. This information could be used by machine learning techniques in order to predict and detect different diseases so that medical interventions take place more rapidly and effectively. Some of the most important diseases, which can be detected through machine learning techniques, are presented below (table 1). 

**Acute kidney injury**

Acute kidney injury (AKI) is a disease that can occur in a few hours to a few days and can lead to kidney failure if not managed properly and the patients would need dialysis for the rest of their lives and they may even die due to kidney failure. However, if this disease is diagnosed soon, it can be rapidly controlled to avoid complications. Artificial intelligence and machine learning techniques can help in fulfilling this objective.

In 2018, a method was proposed, which was based on boosted ensemble of decision trees which was able to detect AKI at time of onset, as well as 12, 24, 48, and 72 hours before the onset of the disease (8). In this study, two databases were used, both of which contained information of patients above 18 years old. Stanford Medical Center dataset contains information of patients in all hospital wards and Beth Israel Deaconess Medical Center contains information of patients in ICU ward collected from MIMIC-III database, which is available. Efficiency of this algorithm was compared with Sequential Organ Failure Assessment (SOFA) method. According to area under the curve (AUC), the accuracy of this method in prediction of AKI at the time of onset is 87%. Additionally, the accuracy of this method for 12, 24, 48, and 72 hours before onset of AKI is 80%, 79%, 76%, and 72%, respectively.

Another study assessed clinical notes in the first 24 hours after admission in intensive care unit (ICU), which is extracted from MIMIC-III dataset (9). By means of natural language processing, meaningful words and representations of concept and embedding were produced and different supervised classifiers such as Multinomial naıve Bayes (MNB), L1-/L2- regularization, SVM, Logistic Regression (LR), Random Forest (RF), Gradient Boosting and Decision Tree (GBDT) and architecture knowledge-guided deep learning were used to design the model. Among these, the highest accuracy belonged to LR with 77.90%.

In one other study in this regard, a model for prediction and detection of AKI in patients above 60 years old was proposed in which collected information of 25521 patients during one year was utilized (10). In this study, five machine learning techniques, including logistic regression, support vector machines, decision trees, naïve Bayes and ensemble were used for disease prediction and detection. These methods were compared with each other and based on AUC; logistic regression was the most accurate method for disease detection with 74% accuracy; while ensemble method was the most accurate for disease prediction with 66% accuracy 24 hours before the onset of the disease.

AKI is a major complication with a very high reported mortality rate for people trapped beneath the rubble following earthquakes. In these cases, AKI can be prevented through rapid and early fluid therapy. This issue is evaluated in a study, which presents two algorithms to help medical staff predict AKI on the first day after a disaster (11). This method is based on biochemical parameters and can be utilized very easily even in disasters. Early prophylactic hydration therapy for patients with kidney disease, who are susceptible to AKI, can reduce its occurrence during natural disasters. Since in these situations, a huge resource for fluid therapy might not be available, it is vital to separate those who are at risk of AKI from those who are not at risk. For this purpose, decision rules were designed by considering Receiver operating characteristic curve and Binary logistic regression, which have a sensitivity of 96.6% and specificity of 95.7% for AKI prediction.

There was another method for AKI risk predication in patients admitted in hospitals. Data of patients were extracted from Electronic Health Record of academic medical centers from 2008 to 2016. Using Gradient Boosting Machine, AKI can be predicted with AUC of 0.73 to 0.97 in different conditions (12).

Deep learning method was deployed in a study on prediction of AKI in ICU ward. This study was conducted on MIMIC-III database and was able to predict AKI with 99.1% accuracy (13).

**Table 1:** Different models on disease prediction

**Disease**	**Algorithm**	**Result**	**Year**
** AKI**	boosted ensembles of decision trees	AUC :0.72- 0.87	2018 (8)
	Logistic regression	AUC: 0.77	2018 (9)
	Gradient Boosting Machine	AUC:0.73-0.97	2018 (12)
	Deep Learning	Accuracy: 99.1%	2018 (13)
	Logistic regression	AUC : 0.74	2016 (10)
	Binary logistic regression	Sensitivity :96.6% Specificity: 95.7%	2008 (11)
**Influenza**	Bayesian classifier (naïve Bayes)	AUC: 0.92-0.93	2015 (15)
	Bayesian network classifiers	AUC: 0.79	2014 (14)
**Sepsis**	Gradient tree boosting	AUC: 0.87-0.92	2018 (18)
	Support Vector Machine	AUC: 0.86	2017 (19)
**COPD and Asthma**	Random forest	C-statistic: 0.84	2018 (20)
	Logistic regression	Accuracy: 89.1%	2017 (21)
	Naive Bayes	Accuracy: 70.7%	2013 (23)
	Tree-based decision model	AUC: 0.83	2010 (22)
**UTI**	Extreme gradient boosting	AUC: 0.90	2018 (17)
**Appendicitis **	Rule base	AP: 0.86	2013 (25)

AKI: acute kidney injury; COPD: chronic obstructive pulmonary disease; UTI: urinary tract infection; AP: average precision and recall.

**Table 2:** Different models for prediction of disposition

**Prediction**	**Algorithm**	**result**	**Year**
Need for admission	XGBoost	AUC: 0.92	2018(29)
Need for admission	gradient boosted machines	AUC:0.85	2018 (30)
ICU Readmission	gradient boosted machine	AUC: 0.76	2018 (33)
In-hospital mortality	LSTM	AUC: 0.94	2018 (37)
Length of ICU stay	logistic regression	AUC :0.93	2018 (37)
Readmission	Random forest	AUC: 0.58	2018 (37)
Need for admission	Naïve Bayes+ logistic regression	AUC: 91.0	2017 (28)
Need for admission	Nu-Support Vector Machine	F-score:0.77	2017 (36)
In-hospital Mortality	Random forest	AUC: 0.86	2016 (34)
Length of stay	logistic regression Random forest	> clinicians	2015 (35)
Need for admission	logistic regression	AUC: 0.80- 0.89	2013 (32)
Need for admission	logistic regression	Specificity=96.8% Sensitivity=33.4%	2011 (31)

ICU: intensive care unit, LSTM: long short-term memory.

**Table 3:** Different models used in machine learning based triage systems

**Algorithm**	**Result**	**Year**
Decision Tree Support Vector Machine	Accuracy=84.0% Accuracy=84.0%	2018 (41)
Random Forest	AUC: 0.73-0.92	2018 (40)
RODDPSO	Mean silhouette value: 0.31	2018 (38)
Naive Bayes classifier Bayesian network	Accuracy: 87.9% Accuracy:86.9%	2008 (39)

RODDPSO: randomly occurring distributed delayed particle optimization.

**Influenza**

Influenza is an infectious disease affecting many people every year and it can cause epidemics due to its contagious nature. Thus, early diagnosis and prediction of influenza can prevent its epidemics and save people’s lives, reduce treatment costs and lessen patient reference to ED.

A study in this regard has proposed an automatic method for influenza diagnosis based on EHR in which symptom extraction, feature selection, and their classification for influenza diagnosis have been performed systematically (14). In fact, natural language processing and different classifiers have been used in a combined manner so that influenza disease can be diagnosed automatically based on EHRs. More accurately, we can state that in this method, free-text reports of ED are processed using different language processing parsers such as Topaz, MedLEE, and an expert to extract the important information about influenza. Then Bayesian network classifier uses expert-defined-BN, BN-EMTopaz, or BN-EM-MedLEE for estimation of influenza probability. Finally, for evaluation of this method, 9 experiments were conducted on three parsers and three classifiers and the results were compared with gold standard. Highest accuracy was achieved when an expert processed the reports and extracted influenza related information and BN-EMTopaz classifiers were used, AUC of which was 0.79 and showed the highest value among the 9 experiments.

Free-text reports in ED can be helpful in early and real-time diagnosis of influenza. In a study, it is reported that influenza is diagnosed in EDs using machine learning techniques based on these reports (15). The data used in this study included 31268 ED reports of 4 hospitals from 2008 to 2011. Then, by means of topaz, a natural language processing tool, features and terms related to influenza were extracted form reports and codified into three categories (acute, non-acute, and missing). Then, 7 classifiers including Naïve Bayes, Bayesian network with the K2 algorithm, Efficient Bayesian Multivariate Classification, Artificial Neural Networks, Logistic Regression, SVM, and Random forests were applied and the results were compared with each other. If there were missed data, Bayesian (naïve base) showed better performance with AUC of about 92-93%.

According to studies conducted to compare different methods of natural language processing in influenza diagnosis, it can be concluded that by using all clinical notes, influenza can be diagnosed with 92.9% accuracy, while when only symptoms reported by the patient is processed, the accuracy of these methods drops to 70.3% (16). 

**Urinary tract infection (UTI)**

Urinary tract infection is a common disease in EDs with a high rate of diagnostic error, since urine culture is not available until 24 to 48 hours after the first visit. Diagnosis and decision making on medication prescription is based on symptoms, physical examination findings, and results of laboratory tests, which can cause overuse of antibiotics and resistance against antibiotics. Previous studies have shown that diagnostic efficacies of laboratory tests and individual predictions are not enough.

A study performed in this regard has proposed some models for prediction of UTI based on machine learning for different patients in ED (17). Data used in this study was collected from clinical reports of health profiles of patients above 18 years old admitted to 4 EDs between 2013 and 2016. Proposed models for disease prediction used 7 machine learning algorithms including Random forest, extreme gradient boosting, adaptive boosting, elastic net, support vector machine, logistic regression, and neural network. Results of this study showed that among the mentioned algorithms, XGBOOT algorithm provided the best efficacy with AUC of 0.90. 

**Sepsis**

Sepsis is a severe infection with a high mortality rate and high therapeutic costs. Consequently, early diagnosis and treatment of sepsis can reduce mortality rate among the patients and reduce therapeutic costs for these patients. A machine learning based method has been proposed for prediction and diagnosis of sepsis, which can improve the treatment procedure of patients (18). Using gradient tree boosting algorithm, three levels of sepsis are detected. Features used in this method include values of 6 vital signs in EDs, general wards, and ICU and eventually, area under the ROC value for sepsis and severe sepsis are 0.92 and 0.87, respectively. 

Another study suggests an automatic system by using machine learning techniques for triage of sepsis patients in ED (19). In this study, a dataset including patient’s information and data including ED triage report note, triage vital signs, and ICD-9-CM codes was extracted from EHR. According to the reported results, it can be concluded that using free-text data from ED triage, as well as structured data such as vital signs and statistical information can significantly improve detection of patients suspected to infection. Two models, bag of words model and topic model were used for representation of free-text data and then an SVM was used to design the predictor model. Applying SVM of bag of words is more effective compared with other methods and AUC value for test and train data are 0.86 and 0.89, respectively. 

**Chronic obstructive pulmonary disease (COPD) and asthma**

Patients with chronic obstructive pulmonary disease and asthma, face the risk of exacerbation of their disease every day. Using special tools when necessary, the probability of exacerbation of the disease can be effectively reduced. By means of machine learning techniques, some methods have been proposed, which can be used for early diagnosis of exacerbation of the disease.

Asthma condition and COPD exacerbation in EDs have been assessed in different studies using different machine learning methods such as Lasso regression, random forest, and boosting, and deep neural network, and some models have been developed based on available data. These methods were compared based on C-statistic index, which showed that Random forest method has the highest efficacy with 84% accuracy (20).

In another proposed algorithm, some features are determined by physicians for both prediction of patient’s triage and diagnosis of disease exacerbation and data are labeled. Then, the algorithm is trained by different methods. According to the obtained result for different classifiers of machine learning, Gradient-Boosted Decision Tree and Logistic Regression had the highest efficacy with an accuracy of 88.1% and 89.1%, respectively (21).

Another study predicts the severity of asthma exacerbation among children in ED of Eastern Ontario Pediatrics Hospital. Data of children aged 1 to 17 years were used and a tree-based decision model was designed for prediction of severity of asthma exacerbation with AUC of 0.83 (22).

Moreover, other machine learning methods have been used to predict this disease. In one of these studies, five different models were designed in this regard and Naïve Bayes model was the most efficient model with 70.7% accuracy. Therefore, this can be used as a complementary model along with the traditional models such as Pediatric Respiratory Assessment Measure (PRAM) score (23). 

**Appendicitis**

Appendicitis is one of the most common causes of abdominal pain in patients referring to ED. A major challenge in diagnosis of appendicitis is wrong diagnosis or delayed diagnosis and perforation. Thus, early and accurate diagnosis of appendicitis is necessary (24).

An automatic system has been designed in which major components are extracted from clinical notes in EDs and laboratory test results. In this model, risk of appendicitis in children is classified to high risk, low risk, and equivocal (25). This method is based on machine learning techniques and natural language processing in both of which structured information derived from EHRs (laboratory results) and clinical notes of ED are used. Firstly, the information about risk of appendicitis is extracted using natural language processing techniques. Then, a rule based method was used to classify appendicitis risk to three classes (high risk, low risk, and equivocal). Finally, efficacy of this method was compared with gold standard method, which is designed manually by physicians. Mean precision and recall of this system were 38% and 86%, respectively.

Electronic surveillance systems can detect the disease faster than diagnosis based systems based on chief complaint of patients. In this regard, a classifier is designed to collect free-text reports about chief complaint of patients in triage to classify patients in one of the 7 following categories: respiratory, botulinic, gastrointestinal, neurologic, rash, constitutional, and hemorrhagic syndromes (26). Final result of the study shows that for most of these syndromes, classification systems can detect nearly half of patients with a specificity more than 90% and positive predictive value of 12 to 44 percent, predicting the related syndrome with an overall accuracy of 92.3% to 99.1% for these 7 syndromes.

**Prediction of disposition and mortality**

Considering the increasing requests, boarding of patients admitted to ED is an issue due to the overcrowding. Prediction of discharge and admission of patients can be performed automatically to improve this process. To achieve this goal, supervised machine learning methods and available health data are used to help in admission of new patients.

Early prediction of admission can speed up allocation of resources and bed to the patient and shorten the boarding times. On the other hand, according to the results, it has been proved that in some cases, nurses are not sure when predicting patient admission and show lower performance compared with machine learning methods (27). Thus, machine learning methods can be time-saving and improve outcomes in medical interventions and patient satisfaction, and reduce hospital costs.

Different models have been proposed for early predicting of patient admission (table 2). In a study in this regard, a model was designed based on combined generative-discriminative approach (28). Number of variables has been reduced by means of naïve Bayes (generative) and then a regression model (discriminative) is applied on the results of the previous model. Using data of available EHRs, this model can predict 73.4% of admissions with a specificity of 90% and 35.4% of admissions with a specificity of 99.5% (AUC=91%) in the first 30 minutes.

Another study suggests a model based on machine learning in which, history of patients is used as well as the information collected in ED triage to predict patient admission or discharge (29). Three binary classifiers, logistic regression, gradient boosting (XGBoost), and deep neural networks, are applied on three kinds of datasets (first dataset only contains patient history, second dataset only contains triage information, and third dataset includes both patient history and triage information). Results show that using patient history in addition to triage information significantly improves efficacy of the prediction and XGBoost classifier provides the best efficacy with AUC of 0.92

In another study, available methods in data mining were applied on electronic system data to form a prediction model for patient admission in ED triage (30). In this study, three algorithms including logistic regression, decision trees, and gradient boosted machines (GBM) have been used and results proved that GBM method provides the best performance with AUC of 0.85 and accuracy of 80.31% compared with the other two methods. Yet, when it comes to interpretability of the data, adopting logistic regression model is a better choice.

Many models developed for prediction of hospital admission in EDs are focused on a special group of patients or patients with special diseases and just a few studies have discussed admission of all patients.

A study suggests a model to predict risk level at the time of admission. In this model, data of all patients with any disease in triage is assessed and data collected from the routine examinations performed at the time of triage is used (31). This model helps nurses in triage to make faster decisions regarding whether the patient should be admitted or not, so that resources are allocated to those who should be admitted and ED crowding is reduced. Logistic regression is used to develop this method with a specificity of 96.8% and sensitivity of 33.4%.

Additionally, result of a study showed that logistic regression method with AUC of 0.80 to 0.89 can be generalized to different hospitals with different number of patients (32).

In some cases, patients are transferred from ICU to general ward and then they are transferred to ICU which can cause problems such as longer ICU stay and increased costs. Also, it can increase mortality rate. This issue is discussed in a study and ICU readmission was predicted by machine learning techniques using data of EHRs (33). Gradient boosted machine is used to design this model and the results are compared with two decision rules Stability and workload index for transfer (SWIFT) score and Modified Early Warning Score (MEWS). It can be concluded from reported results that the proposed method with AUC of 0.76 is significantly better than MEWS method with AUC of 0.65 and SWIFT method with AUC of 0.58.

Machine learning methods can also be helpful in prediction of patient mortality. Analyses and predictions performed in ED are often limited to clinical decision rules (CDRs), which use simple heuristics and scoring systems. A major problem of CDRs is that they are not generalizable and cannot be updated using new data. But, new techniques are based on machine learning and can use many variables from EHRs. Mortality of patients with sepsis in ED is predicted using a machine learning based method (34). A random forest model was developed using data of EHRs and was compared with models developed based on regression tree (CART) and logistic regression models, where it showed better efficacy with AUC of 0.86. Moreover, machine learning approach can better predict mortality rate of patients with sepsis compared with available CDRs and traditional data analysis techniques.

Also, machine learning can be used to predict hospital length of stay so that patients can be prioritized for discharge. Logistic regression and random forest method have been used in a study (35). Comparison of these methods with prediction of hospital staff revealed that both methods have higher sensitivity and lower specificity.

Several other studies have been conducted in prediction of admission or discharge of patients using different machine learning techniques. In one of these studies, prediction is performed by text mining of clinical reports by applying different algorithms. Comparison of these methods showed that, Nu-Support Vector Machine provides the best performance with F1 score of about 0.77 in comparison to other algorithms (36).

Another study has evaluated patient admission, in hospital patient mortality, and length of stay on MIMIC-III database, separately. Different classic machine learning methods such as SVM, LR, MLP, Random forest, and Gradient Boost classifier and sequential models such as LSTM and CNN-LSTM have been used and the results are compared (37). In terms of patient mortality prediction, among classic models, MLP method with AUC of 0.85 and among sequential models, LSTM model with AUC of 0.94 showed the highest accuracies. In terms of length of stay prediction, among classic models, logistic regression method with AUC of 0.93 and among sequential models, LSTM model with AUC of 0.88 provided the best efficacies. Finally, in terms of patient admission, Random forest and LSTM models had provided the best results with AUC of 0.58.

**Machine learning based triage systems**

Since EDs are overcrowded, the main problem in triage is to classify patients based on disease severity with a fast and accurate method to provide best services. Using machine learning techniques can improve pace and efficacy of patient management in triage compared with traditional methods. Studies performed in recent years are discussed in the following paragraphs (table 3).

Recently, a study has been conducted to suggest a method for proper classification of patients in triage into 5 groups based on disease severity (38). A new algorithm, namely Randomly Occurring Distributed Delayed Particle Swarm Optimization (RODDPSO), is proposed in this study, which is based on PSO evolutionary algorithms and is a method for clustering ED data suggested for patient classification. Efficacy of this method was finally evaluated based on mean silhouette value with two clustering algorithms K-means and FCM. Reported results showed that RODDPSO with a value of 0.31 for the mentioned index has a higher efficacy compared with the other two methods. Instead of using traditional protocols in triage, which are mostly based on disease symptoms, clinical decision support systems (CDSSs) may be used.

CDSSs have been evaluated in a study for triage assistance using machine learning algorithms, which directly learn the model form data instead of an expert (39). There are different approaches for designing a model and the method should be chosen in a way that the result can be interpreted by physicians. For instance, decision trees and Bayesian networks are appropriate methods. Furthermore, types of information used to develop the model are also important. Expert opinion or data can be used as information resources. Studies suggest that using data and machine learning methods acts better compare with expert opinion in models. Also, Bayesian networks are more interpretable compared with rule-based models such as decision tree. Thus, developing models based on these methods can provide higher accuracy compared with other models.

Bayesian network algorithms achieved accuracy of 87.9% and 86.9% by using Naïve Bayes classifier and K2 algorithm, respectively, which are much higher compared with Decision tree method.

Usually, standards of ED triage mostly depend on individual diagnosis and they have limited ability in identifying high risk patients. A method of assessment based on electronic triage using machine learning techniques is proposed in a study, which can predict high risk patient and separates patients properly (40). To be more accurate, this study classifies ESI level 3 patients so that prediction results can be more easily analyzed in triage. In this study, three decision tree learning models or in other words, random forest is used to predict intensive care, emergency procedure, and hospital admission for ED patients. Finally, the output of this model for each patient is one of these three categories. Results of this prediction has an AUC ranging from 0.73 to 0.92. This method classifies ESI level 3 patients more accurately. 

Another study on electronic triage of ED patients has proposed models with supervised machine learning algorithms and compared them (41). Classifiers such as Naive Bayes, Support Vector Machine, Decision Tree, and Neural Network have been deployed, among which Support Vector Machine and Decision tree provided highest efficacy with an accuracy of 84%. Another study proposed a calculative algorithm using fuzzy logic and decision tree to classify patients in ED triage (42). 

## Conclusion

Abilities of artificial intelligence and machine learning techniques can be used in medicine, especially in emergency medicine and in some important issues including disease prediction, admission or discharge prediction, and patient triage. By early prediction and diagnosis of high risk diseases such as AKI, sepsis, pneumonia, and contagious diseases such as influenza, necessary interventions can be performed more rapidly in ED to prevent multiple disease progression complications. In this regard, different machine learning algorithms such as Logistic regression, Bayesian network, deep learning etc. have been deployed, which generally have shown high accuracy ranging from 70% to 90%. Additionally, these algorithms and other methods can be helpful in prediction of patient admission and improve patient triage.

## Conflict of interest:

The authors have no conflict of interest to declare.

## Support:

None.
